# A Fault Diagnosis Method for Oil Well Electrical Power Diagrams Based on Multidimensional Clustering Performance Evaluation

**DOI:** 10.3390/s25061688

**Published:** 2025-03-08

**Authors:** Xingyu Liu, Xin Meng, Ze Hu, Hancong Duan, Min Wang, Yaping Chen

**Affiliations:** 1 School of Electrical Information, Southwest Petroleum University, Chengdu 610500, China; 202222000120@stu.swpu.edu.cn (X.L.);; 2 School of Computer, University of Electronic Science and Technology of China, Chengdu 611731, China

**Keywords:** beam-pumping system, electrical parameters, FCM clustering algorithm, cluster validity function

## Abstract

In oilfield extraction activities, traditional downhole condition monitoring is typically conducted using dynamometer cards to capture the dynamic changes in the load and displacement of the sucker rod. However, this method has severe limitations in terms of real-time performance and maintenance costs, making it difficult to meet the demands of modern extraction. To overcome these shortcomings, this paper proposes a novel fault detection method based on the analysis of motor power parameters. Through the dynamic mathematical modeling of the pumping unit system, we transform the indicator diagram of beam-pumping units into electric power diagrams and conduct an in-depth analysis of the characteristics of electric power diagrams under five typical operating conditions, revealing the impact of different working conditions on electric power. Compared to traditional methods, we introduce fourteen new features of the electrical parameters, encompassing multidimensional analyses in the time domain, frequency domain, and time-frequency domain, significantly enhancing the richness and accuracy of feature extraction. Additionally, we propose a new effectiveness evaluation method for the FCM clustering algorithm, integrating fuzzy membership degrees and the geometric structure of the dataset, overcoming the limitations of traditional clustering algorithms in terms of accuracy and the determination of the number of clusters. Through simulations and experiments on 10 UCI datasets, the proposed effectiveness function accurately evaluates the clustering results and determines the optimal number of clusters, significantly improving the performance of the clustering algorithm. Experimental results show that the fault diagnosis accuracy of our method reaches 98.4%, significantly outperforming traditional SVM and ELM methods. This high-precision diagnostic result validates the effectiveness of the method, enabling the efficient real-time monitoring of the working status of beam-pumping unit wells. In summary, the proposed method has significant advantages in real-time performance, diagnostic accuracy, and cost-effectiveness, solving the bottleneck problems of traditional methods and enhancing fault diagnosis capabilities in oilfield extraction processes.

## 1. Introduction

The rod pumping system stands as the primary equipment in oilfield development for oil extraction. It not only plays a crucial role in the production process but also serves as an essential window into the dynamic information of underground reservoirs [1]. Consequently, real-time evaluation technology for rod pumping systems holds significant theoretical importance and practical value [2]. However, monitoring the operational status of rod pumping equipment poses substantial challenges due to the complex and harsh working conditions thousands of meters underground, where sensors for load and displacement must be deployed [3]. Traditional methods [4,5,6], such as on-site dynamometer cards, often fail to accurately and promptly reflect the working conditions of the pumping unit. This inadequacy can severely affect oilfield production efficiency and may lead to mechanical equipment damage [7].

In contrast, measuring the electrical parameters of the motor driving the pumping unit offers several advantages as follows: convenience, high accuracy, reliability, and cost-effectiveness [8]. These parameters are crucial for realizing the long-term online monitoring of the pumping unit system and present obvious application benefits. Despite these advantages, evaluation methods based on electrical parameters have not been deeply studied due to the lack of comprehensive models and effective data processing techniques [9].

In recent years, although some scholars have attempted to use motor power curves for fault diagnosis, most methods are limited to the analysis of single features or have limitations in data processing, failing to meet the real-time monitoring needs in big data environments. Moreover, existing classification and diagnosis methods lack effective evaluation mechanisms when handling multi-dimensional electrical parameter information, affecting the accuracy and reliability of the diagnostic results.

Therefore, to overcome the limitations of the existing fault diagnosis methods for pumping units based on electrical parameters, we propose a novel approach that combines multi-feature extraction with a fuzzy C-means clustering effectiveness evaluation function. Through this method, we construct a mathematical model to transform the dynamometer card into an electrical power diagram. We then extract multiple features, including the upstroke and downstroke power, cyclic power deviation, cragginess, and marginal spectral characteristics from the motor power curves under different operational conditions. Finally, we employ a novel fuzzy C-means clustering effectiveness function to classify and recognize the operational states of the pumping unit. The contributions of this study include the following: (1) We develop a new mathematical model to transform the dynamometer card into an electrical power diagram, facilitating more accurate and effective feature extraction and analysis.

(2) To address the issue of high computational complexity caused by the low utilization of feature maps in input features, we introduce fourteen new features for the electrical parameters, encompassing multidimensional analyses in the time domain, frequency domain, and time–frequency domain, significantly enhancing the richness and accuracy of feature extraction. By performing multi-feature extraction on electrical parameters, we uncovered a significant amount of fault information, solving the technical bottlenecks in current pumping unit fault diagnosis. This approach significantly reduces algorithm complexity and greatly enhances the accuracy of working condition identification.

(3) We proposed a new fuzzy C-means clustering effectiveness evaluation function, which employs a multidimensional clustering performance evaluation approach to assess the performance of clustering algorithms, overcoming the limitations of traditional clustering algorithms in terms of accuracy and the determination of the number of clusters, aiming to improve classification accuracy in pumping unit fault diagnosis.

This paper is structured as follows. Section 2 summarizes and reviews the relevant research in the field of fault diagnosis for pumping units. Section 3 employs dynamic mathematical modeling of the pumping system, calculates sample motor power curves under different operating conditions, analyses the characteristics of the electric power diagrams under five typical operating conditions, and extracts 14 features. Section 4 puts forward a novel fuzzy C-clustering validity function model for classification. Section 5 demonstrates the efficacy of feature extraction in this paper through field validation. Section 6 presents a discussion and summary.

## 2. Related Work

The intelligent fault diagnosis of pumping unit systems based on electrical parameters is an emerging and challenging topic in the oil and gas industry. Scholars have proposed numerous methods for diagnosing pumping unit faults, which can be categorized into numerical simulation methods, machine learning methods, and fuzzy theory methods. The details of these methods are described below.

### 2.1. Numerical Simulation Methods

In recent years, numerous scholars have explored the fault diagnosis of beam-pumping unit systems based on electrical parameters. Early work by [10] proposed a method for calculating the schematic diagram based on the motor power curve. However, due to the torque coefficient approaching zero near the upper and lower dead points, the calculated figure was not closed, resulting in significant errors. They further proposed a method to accurately identify the upper and lower deadspots, and they smoothed the motor power outliers near the deadspots to improve the accuracy. Nonetheless, this identification and smoothing method had limited practicality and faced operational challenges. The authors in [11] advanced this area by using motor power curves to analyze the efficiency of rod pumping systems more accurately and effectively. Similarly, ref. [12] proposed a soft measurement method that obtains an oscillogram using the motor input electrical parameters and the inclination of the traveling beam as inputs. Although these studies made progress in using motor power for well monitoring, the effectiveness of the motor power in pumping machine operations has not been fully analyzed.

### 2.2. Machine Learning Methods

Machine learning techniques have also been applied to this problem. The quality of feature extraction directly affects the performance and accuracy of machine learning algorithms. The authors in [13] conducted a frequency domain analysis of power curves using wavelet transforms for diagnosing rod pump failures. However, they noted that the motor power curve cannot be adequately characterized by frequency domain features alone. To address the limitations of traditional manual calculation and feature extraction methods, especially given the massive data generated by oilfield pumping machines, the authors in [14] established a mathematical model to convert the mechanical power diagram of pumping rods into an electrical diagram. Despite these advancements, traditional diagnostic methods struggled to adapt to large-scale data environments. The authors in [15] employed motor power data and the XGBoost algorithm to diagnose the working states of sucker rod pumps. However, the classification results were inaccurate because sand production—which can produce curves similar to gearbox faults—was not considered. To improve fault detection, the authors in [16] transformed the motor power curve into an oscillogram and extracted its features. They then used a Hidden Markov Model for fault detection. While these studies advanced the extraction of electrical parameter features, they lack a comprehensive multi-dimensional analysis of electrical parameter information.

### 2.3. Fuzzy Theory Methods

The application of fuzzy mathematics theory in the diagnosis of pumping unit failures has yielded encouraging outcomes in recent years. The determination of membership functions is fundamental to the application of fuzzy theory in studying fuzzy problems. In [17], a rough classifier based on the rough set theory was used to diagnose and identify five types of failures in rod pumps. Addressing the issue of supervised learning methods being highly dependent on training samples, the authors in [18] proposed an unsupervised learning approach for downhole fault diagnosis, introducing an improved fuzzy iterative self-organizing data analysis technique with “merging” and “splitting” mechanisms. In 2016, the authors in [19] proposed a method for diagnosing faults in sucker rod pumps based on wavelet moment features and fuzzy kernel clustering algorithms. More recently, ref. [20] introduced a methodology for the expeditious annotation of pumping well dynamometer cards based on K-means clustering. While the aforementioned studies have made notable strides in applying fuzzy mathematics theory to pumping unit fault diagnosis, there is a paucity of research on the reasonable and accurate evaluation of classification performance.

In summary, although significant progress has been made in diagnosing pumping unit faults using electrical parameters and fuzzy mathematics, the existing methods often face limitations in accuracy, practicality, or scalability. There remains a need for comprehensive models and effective data processing methods that leverage multi-dimensional electrical parameter information for more accurate and practical fault diagnosis in pumping units. This paper aims to fill this gap by introducing a novel approach that combines multiple electrical parameters’ feature extraction with an improved fuzzy C-means clustering effectiveness evaluation function.

## 3. Motor Power Sample Generation and Feature Extraction

The balance of the traveling beam-pumping unit can be directly gauged by the electric power diagram; however, there is currently no established set of electric power diagrams that can be used as a reference point for typical working conditions. This lack of a mature sample library of electric power diagrams hinders the application of electric power diagrams in the diagnosis of working conditions. In contrast, schematic diagrams have already accumulated a mature set of diagrams over time. In the preceding period, the mechanical mechanism of the traveling beam-pumping unit was analyzed, and a mapping model of the electric power diagrams and schematic diagrams was established. This has the potential to provide data and technical support for the diagnosis of working conditions based on electric power diagrams. In this section, an electric power generation model is constructed, and the motor power under five operating conditions is obtained by converting the schematic diagram. This is used to study the motor power characteristics under different operating conditions.

### 3.1. Mathematics of Beam-Pumping System Dynamics Modeling

**Figure 1:**
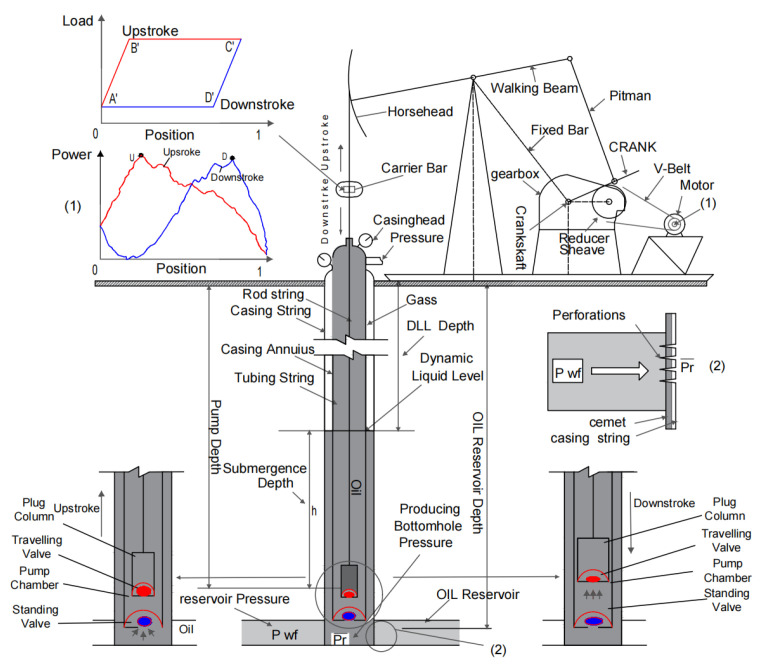
The diagram of the schematic of sucker rod pump: (1) electrical power diagram, (2) the perforation holes.

This section primarily focuses on the theoretical modeling and simulation of the traveling beam-pumping unit system, detailing the method for calculating the electric power curve derived from the dynamometer cards. The electrical parameter simulation model is employed to simulate and analyze the surface machinery of traveling beam-pumping unit wells, as depicted in Figure 1.

**Figure 2:**
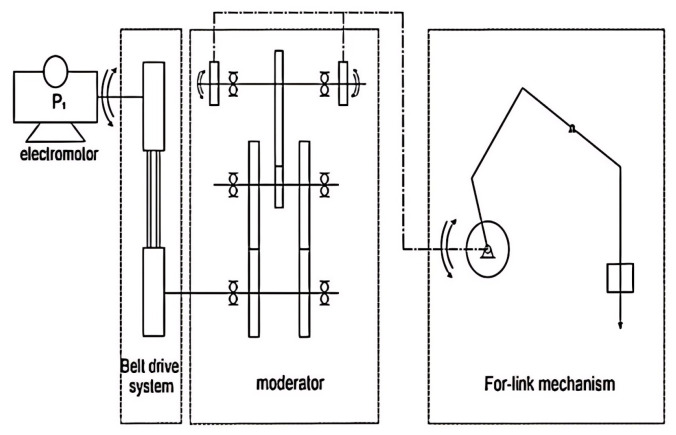
Simplified model of pumping unit.

**Figure 3:**
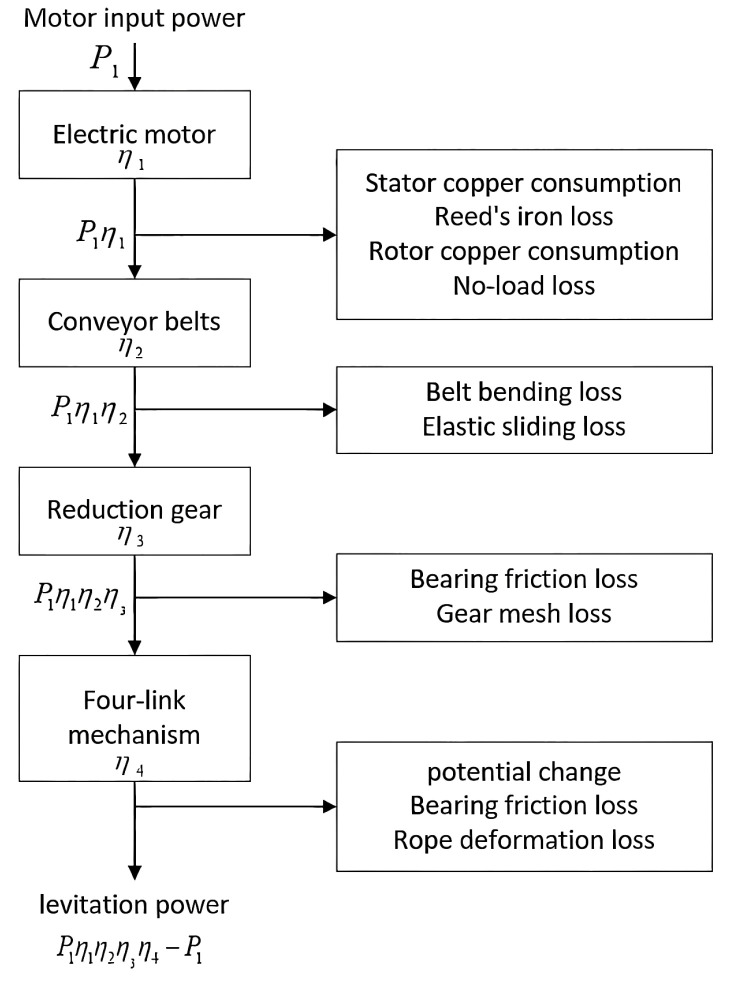
Motor power flow diagram.

This machinery primarily includes the beam-pumping unit, the reducer, and the motor. The pumping system, driven by the asynchronous motor, comprises the motor, the reducer, the four-link mechanism, and the oil pumping device. The torque output from the motor is transmitted to the gearbox via the belt transmission system, where the four-link mechanism converts the rotary motion from the gearbox into the up-and-down reciprocating motion of the suspension point. The simplified model of the entire surface system of the traveling beam-pumping unit is illustrated in Figure 2. To fulfill the system’s functional requirements, energy must be converted from electrical to mechanical form, as depicted in the motor power flow in Figure 3.

#### 3.1.1. Studying Speed Fluctuations in Motion Accuracy Analysis

**Figure 4:**
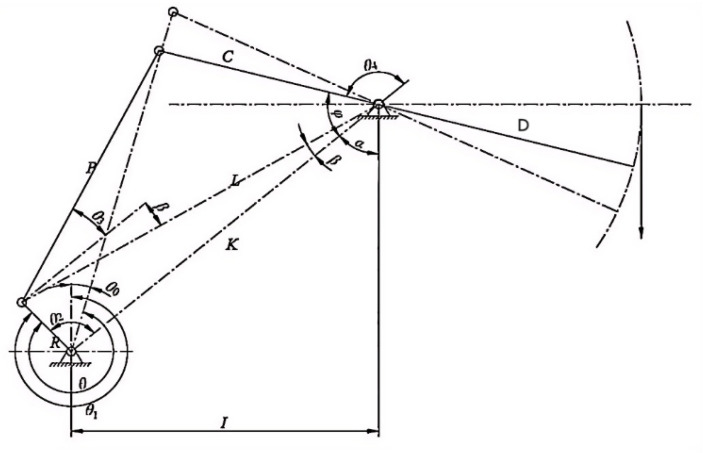
Schematic diagram of the beam-pumping unit mechanism.

The schematic diagram of the motion mechanism of a conventional beam-pumping unit is shown in Figure 4, and the key parameters in the figure are listed in Table 1. The pumping machine kinematic parameters related to the pumping machine ground schematic are suspension point displacement *s*, suspension point velocity *v*, suspension point acceleration *a*, the torque factor $\bar{TF}$, the angle of the traveling beam to the horizontal line δ, crank angle θ, and the number of strokes of the oil flushing machine *n*, etc. Among them, *s*, $\bar{TF}$, and δ are only related to the motion position of the pumping machine. These parameters depend on the crank angle θ, which is a function of both the crank angle itself and the structural parameters of the pumping unit. Specifically, they can be expressed as follows:

(1) $$f\left(\theta ,R,C,L,K,P\right)=\left(s,\bar{TF},\delta \right)$$

#### 3.1.2. Electrical Parameter Simulation Model Based on the Polished Rod Dynamometer Card

The torque generated on the crankshaft by the structural weight of the pumping unit, excluding the overhanging point load, is denoted as $T_{0}$ [21], as follows:

(2) $$T_{0}=T_{y}+T_{r}+T_{B}$$

where $T_{0}$ is the total torque; $T_{y}$ is the traveling beam equilibrium torque; $T_{r}$ is the crank balancing torque; and $T_{B}$ is the unbalanced weight balancing torque.

The torque generated on the crankshaft by the suspension point load is referred to as the pure rod load torque $T_{wn}$, expressed as follows:

(3) $$T_{wn}=W\bar{TF}$$

(4) $$\bar{TF}=\frac{\;\mathrm{DRsin}\;\theta_{2}}{\sin\left(\pi -\theta_{0}-\psi \right)}$$

where *W* is the suspension point load, $\bar{TF}$ is the torque factor, and $\bar{TF}$ can be derived from Equations (5)–(12), as follows:

(5) $$\alpha =\sin^{-1}\left(\frac{I}{K}\right)$$

(6) $$\beta =\sin^{-1}\left(\frac{R}{L}\sin\theta_{2}\right)$$

(7) $$L=\sqrt{R^{2}+K^{2}-2RK\cos\theta_{2}}$$

(8) $$\theta_{2}=2\pi -\theta_{0}-\theta +\alpha$$

(9) $$\theta_{3}=\cos^{-1}\left(\frac{P^{2}+L^{2}-C^{2}}{2PL}\right)-\beta$$

(10) $$\theta_{4}=\cos^{-1}\left(\frac{L^{2}+C^{2}-P^{2}}{2PL}\right)-\beta$$

(11) $$\chi =\cos^{-1}\left(\frac{C^{2}+P^{2}-L^{2}}{2PL}\right)$$

(12) ψ=χ+β

Considering the direction of torque, the total torque on the crankshaft is $T_{n}$, expressed as follows:

(13) $$T_{n}=T_{wn}-T_{0}=T_{wn}-T_{y}-T_{r}-T_{B}$$

Ignoring the crank balance inertia torque when the following holds:

(14) $$T_{n}^{\prime }=T_{n}$$

When the motor input power is below a certain threshold, the transmission efficiency of the motor, belt, and reducer increases as the motor input power increases (assuming the frequency converter remains unchanged). Consequently, the total transmission efficiency of the motor-belt-reducer system becomes a function of the motor input power, as follows:

(15) $$\eta_{\mathrm{total}\;}=f\left(P_{1}\right)$$

Among them, $\eta_{\mathrm{total}\;}$ can be obtained by testing the motor power at no load or by comparing the measured oscillogram with the motor input power.

By processing data that measures the real-time speed of the motor, we can also calculate the real-time number of revolutions of the motor. Since the transmission ratio of the belt reducer is fixed, each revolution of the motor corresponds to a crank rotation angle of $\frac{6.28}{i_{\mathrm{belt}\;}i_{\mathrm{gearbox}\;}}$rad. The crank angle is expressed as follows:

(16) $$\theta =\frac{2m_{0}\pi }{i_{\mathrm{total}\;}}$$

where $m_{0}$ represents the accumulated revolutions of the motor from the pumping unit’s lower dead center; and $i_{\mathrm{total}\;}$ is the total transmission ratio of the belt and the reducer.

Considering the instantaneous transmission efficiency of the four-link mechanism, the output shaft of the reducer provides the following torque:

(17) $$\begin{array}{c} T_{n}^{\prime }=\left[W+\left(\frac{a}{g}-1\right)B\cos\delta \right]\bar{TF}\eta_{b}^{m}\;-\left(Q_{el}R_{e1}+Q_{e2}R_{e2}\right)\sin\left(\theta -\tau \right) \end{array}$$

where $Q_{el}$ and $Q_{e2}$ are the total weight of the crank and the balance block on both sides of the pumping unit; $R_{e1}$ and $R_{e2}$ are the distance between the center of gravity of the cranks and their balance blocks on both sides of the pumping unit and the crankshaft; τ is the phase angle of the pumping unit balance; $\eta_{b}^{m}$ is the efficiency of the four-link mechanism; *m* is the efficiency index; when $\bar{TF}$ > 0, m = −1; and when $\bar{TF}$ < 0, m = 1.

Considering the motor speed fluctuation and drive system efficiency, the output torque of the reducer is calculated from the motor input power as follows [22]:

(18) $$T_{n}^{\prime }=\frac{i_{\mathrm{total}\;}}{n_{\mathrm{motor}\;}}9554P_{1}{\left(\eta_{1}\eta_{2}\eta_{3}\right)}^{m}$$

where $P_{1}$ is the inverter input power; $\eta_{1}\eta_{2}\eta_{3}$ represent the motor’s instantaneous efficiency, belt instantaneous efficiency and gearbox instantaneous efficiency; *m* is the efficiency index; when $T_{n}^{\prime }$ > 0, *m* = 1; when $T_{n}^{\prime }$< 0, *m* = −1; $n_{\mathrm{motor}}$ is the instantaneous motor speed; and $i_{\mathrm{total}\;}$ is the total transmission ratio of the belt and the reducer.

The relationship between the motor input power and the suspension point load is expressed as follows:

(19) $$\begin{array}{cc} \; & P_{1}=\frac{n_{\mathrm{motor}\;}\left\{W-\left[\left(Q_{e1}R_{e1}+Q_{e2}R_{e2}\right)\sin\left(\theta -\tau \right)\right]\eta_{b}^{k_{2}}\right\}}{9554{\left(\eta_{1}\eta_{2}\eta_{3}\right)}^{k_{1}}i_{\mathrm{total}\;}\cdot \eta_{b}^{k_{2}}}-\\ \; & \frac{n_{\mathrm{motor}\;}\left[\frac{Q_{y}K_{y}\cos\delta }{A}\left(1-\frac{K_{y}a}{gA}\right)\bar{TF}+B\cos\delta \left(1-\frac{a}{g}\right)\bar{TF}\right]}{9554{\left(\eta_{1}\eta_{2}\eta_{3}\right)}^{k_{1}}i_{\mathrm{total}\;}\cdot \eta_{b}^{k_{2}}} \end{array}$$

where $T_{m}$ is the motor’s electromagnetic torque, when $T_{m}$ > 0, $k_{1}$ = 1; and when $T_{m}$ < 0, $k_{1}$ = −1. When $\bar{TF}$ > 0, $k_{1}$ = 1; when $\bar{TF}$ < 0, $k_{1}$ = −1.

### 3.2. Motor Power Curve Analysis

In this section, according to the constructed electric power generation model, the dynamometer cards under five typical working conditions are converted, and the basic parameters of the five wells are shown in Table 2. The five working conditions chosen for analysis are frequently encountered and have a direct impact on the system’s performance and reliability in real-world operations. The “normal” condition signifies optimal performance, while conditions such as “insufficient liquid supply” and “gas-affected” are common operational challenges that affect the pump’s efficiency. “Gearbox failure” and “oil sands” are critical failure modes that can lead to severe damage if not detected early. These conditions were selected due to their relevance in typical oilfield pumping operations, where timely diagnosis can help prevent costly repairs and downtime.

**Figure 5:**
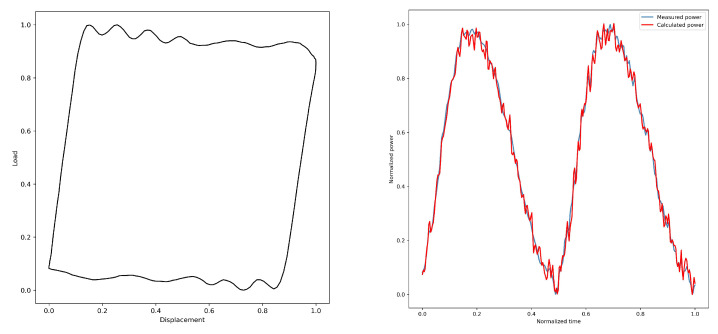
1#—Normal.

**Figure 6:**
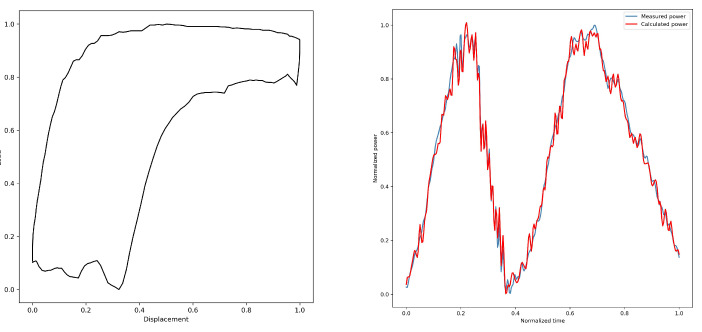
2#—Gas-affected.

**Figure 7:**
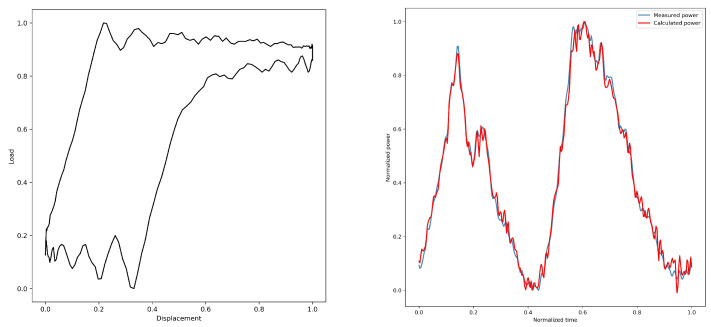
3#—Insufficient liquid supply.

**Figure 8:**
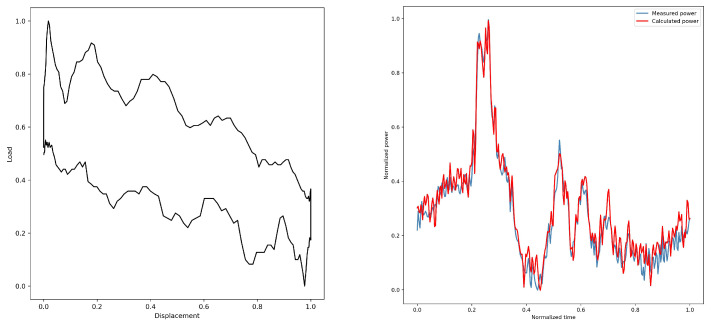
4#—Oil sands.

**Figure 9:**
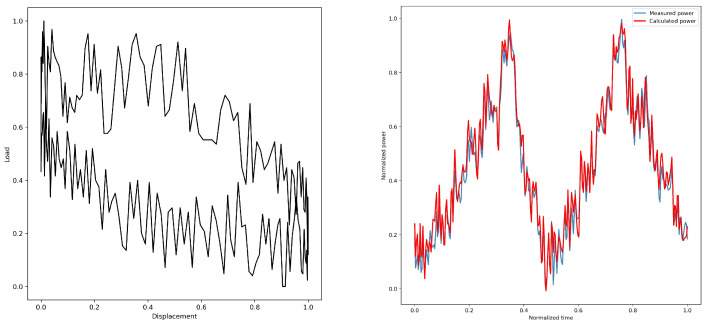
5#—Gearbox failure.

By converting the dynamometer cards, the electric power diagrams under their corresponding working conditions are obtained from Figure 5, Figure 6, Figure 7, Figure 8 and Figure 9. As listed in Table 3, we can find that the calculated electric power and the measured electric power are well fitted within the allowable error range, and the converted electric motor power curves in this paper can better characterize the measured electric motor power curves. This is used to study the motor power characteristics under different operating conditions.

**Figure 10:**
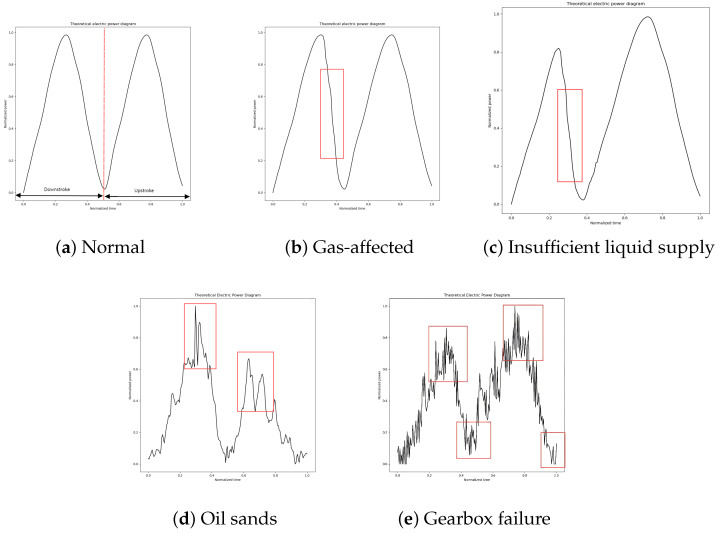
Theoretical electric power diagrams: (**a**) normal. (**b**) gas-affected. (**c**) insufficient liquid supply. (**d**) oil sands. (**e**) gearbox failure.

When the pump is operating normally, it means that all parts of the pumping unit system are functioning correctly, the well has sufficient fluid supply, and the submergence is deep. Additionally, the pump valves do not leak, and the pump operates with high efficiency, resulting in a relatively high oil production under normal operating conditions. The motor power curve is shown in Figure 10a, where the peak power values during the upstroke and downstroke are identical and the trends of the power curve are similar. At the end of loading and unloading during the downstroke, the power curve shows a distinct inflection point. The slope of the curve is steep during the loading and unloading segments, while the slope is smaller during the steady load segment.

The electric power diagram of the gas-affected condition is shown in Figure 10b. In the upstroke, due to the delayed opening of the standing valve, the load line of the electric power diagram changes gently, and the corresponding power curve does not have an obvious inflection point at the end of loading; in the downstroke, due to the delayed opening of the traveling valve, the unloading becomes slow, and the inflection point of the power curve is shifted to the left relative to the normal working condition. The peak power is the same for the upper and lower strokes.

The insufficient fluid supply electric power diagram is shown in Figure 10c. The trend of the power curve change in the upstroke is consistent with the normal working condition, and the power in the start-up section is lower and changes are smaller, which is because the polished load cannot be unloaded in time in the downstroke until unloading starts and the power curve rises rapidly. At the start and end points of unloading, the power curve shows an inflection point, and the time corresponding to this inflection point shifts to the left compared to under normal operating conditions. The time corresponding to the inflection point is shifted to the left relative to the normal working condition. If the liquid supply is seriously insufficient, the power in the downstroke cannot reach the maximum value under normal working conditions, so the power peak in the downstroke is slightly smaller than that in the upstroke.

The electric power diagram of sand production is shown in Figure 10d. The uncertainty of sand production leads to many spikes in the electric power curve.

The gearbox failure electric power diagram is shown in Figure 10e. Similar to the sand production power curve, there are many “spikes”, but the difference is that, at this time, the amplitude of the “spikes” is enhanced and there is periodic occurrence.

### 3.3. Feature Extraction Methods

#### 3.3.1. Time Domain Analysis

Through the analysis in this paper, different operating states correspond to different motor power curves; therefore, the diagnosis of pumping machine operating conditions can be realized by varying electric power over time. Nine features are defined in the time domain as follows:

1. Upstroke power. The sum of the work done by the motor during the upstroke of the pumping unit operation is called the upstroke work, expressed as follows:

(20) $$\psi_{1}=E_{\mathrm{upstroke}\;}=\int_{0}^{t_{\mathrm{top}.\;\mathrm{dead}.\;\mathrm{center}\;}}p\left(t\right)dt$$

2. Downstroke power. The sum of the work done by the motor during the downstroke of the pumping unit operation is called the downstroke work. In fault conditions, the electric power diagram tends to show noticeable fluctuations in the downstroke curve, expressed as follows:

(21) $$\psi_{2}=E_{\mathrm{downstroke}\;}=\int_{t_{\mathrm{downdead}.\;\mathrm{center}\;}}^{T}P\left(t\right)dt$$

3. Cycle work deviation. Under normal operating conditions, the upstroke and downstroke work of a beam-pumping unit are generally balanced. However, when a fault occurs, a significant disparity in work between the two strokes may emerge, expressed as follows:

(22) $$\psi_{3}=e=E_{\mathrm{upstroke}\;}-E_{\mathrm{downstroke}\;}$$

4. Power balance degree:

(23) $$\psi_{4}=B=\frac{{\bar{E}}_{\mathrm{upstroke}\;}}{{\bar{E}}_{\mathrm{downstroke}\;}}\times 100\%$$

${\bar{E}}_{\mathrm{upstroke}\;}$: average upstroke power; ${\bar{E}}_{\mathrm{upstroke}\;}$: average downstroke power. Generally, the balance of a pumping unit system is determined by whether the degree of balance falls between 80% and 110%. It is commonly accepted that if the degree of balance exceeds 1.1, the pumping unit is underbalanced, and if the degree of balance is less than 0.8, the unit is overbalanced.

5. First-order moment statistics: The mean, quantile and triple mean. The mean describes the centralized location of the order data and is considered a stable component of the signal. The sample mean is generally calculated according to the following formula:

(24) $$\psi_{5}=\bar{X}=\frac{1}{N}\sum_{i=1}^{N}x_{i}\left(t\right)$$

Sort the data from smallest to largest in a new order as $x_{\left(1\right)}\leq x_{\left(2\right)}\leq x_{\left(3\right)}\leq \ldots \leq x_{\left(n\right)}$. Let 0≤p≤1, then, the P-quartile is expressed as follows:

(25) $$\psi_{6}=M_{P}=\left\{\begin{array}{cc} x_{\left([np]+1\right)} & ,nP\;\mathrm{is}\;\mathrm{not}\;\mathrm{an}\;\mathrm{integer}\;\\ \frac{1}{2}\left(x_{\left(np\right)}+x_{\left(np+1\right)}\right) & ,nP\;\mathrm{is}\;\mathrm{an}\;\mathrm{integer}\; \end{array}\right.$$

where [nP] stands for *n* multiplied by *P* and then rounded.

The triple mean is the weighted average of the upper quartile, median and lower quartile, calculated as follows:

(26) $$\psi_{7}=\hat{M}=\frac{1}{4}M_{0.25}+\frac{1}{2}M_{0.5}+\frac{1}{4}M_{0.75}$$

6. Second-order moment statistics: The mean square value and variance. The mean square value can be used to represent the energy of a vibration signal when describing the vibration signal, expressed as follows:

(27) $$\psi_{8}=X_{\mathrm{rms}}^{2}=\frac{1}{N}\sum_{i=1}^{N}x_{i}^{2}\left(t\right)$$

Variance, also known as the second-order central moment, is a statistic that describes the relative dispersion of data values:

(28) $$\psi_{9}=s^{2}=\frac{1}{N-1}\left(\sum_{i=1}^{N}x_{i}^{2}\left(t\right)-2\bar{X}\sum_{i=1}^{N}x_{i}\left(t\right)+N\bar{X}\right)$$

#### 3.3.2. Time–Frequency Domain Analysis

The shape characteristics of the electric power diagram curve of the pumping machine can accurately show the working state of the pumping machine under certain faults, but this also has certain limitations. Sand production failure occurs when sand particles enter the pump, creating significant resistance to the movement of the plunger. This resistance leads to the vibration of the pumping rod, which is also reflected as strong vibrations in the electric power diagram. Reduction gearbox failure primarily involves broken teeth on the gears. Such gear damage can cause violent impacts, leading to large high-frequency fluctuations in motor power. During this time, the power diagram’s frequency curve becomes rich with information. A typical power diagram is shown in Figure 10d,e. At this point, these two conditions cannot be effectively identified solely through the changes in the power diagram during the upstroke and downstroke. Therefore, it is necessary to analyze the time–frequency information of the power diagram to accurately diagnose these conditions. To address this, feature extraction of the electropower diagram using the improved Hilbert–Huang transform [23,24] is explored to enhance the characterization of the electropower diagram.

The improved Hilbert–Huang Transform (HHT) consists of the following two components: the CEEMDAN decomposition and the Hilbert transform. HHT is a method used to analyze non-linear and non-stationary signals, which are commonly encountered in real-world fault diagnosis scenarios. The CEEMDAN decomposition enhances the traditional Empirical Mode Decomposition (EMD) algorithm by addressing the issue of mode mixing through the addition of adaptive Gaussian white noise and the iterative averaging of the primary Intrinsic Mode Function (IMF) components. This refinement improves the accuracy and stability of signal decomposition, allowing for the more precise extraction of features from complex signals. The Hilbert transform is then applied to the decomposed IMFs to obtain instantaneous frequency and amplitude information, which can be used for further analysis and fault detection. By combining these two components, HHT offers a powerful tool for analyzing signals that exhibit time-varying and frequency-varying characteristics, such as those found in oilfield pumping systems.

The specific steps of CEEMDAN decomposition are as follows:

First, add Gaussian white noise components with zero mean and unit variance, decomposed from the EMD, to the original signal x. The local mean decomposition sequence is as follows:

(29) $$x^{i}\left(t\right)=x\left(t\right)+\varepsilon_{0}E_{1}\left[\xi^{i}\left(t\right)\right]$$

Obtain the local mean signal of the decomposed sequence $M\langle x\left(t\right)+\varepsilon_{0}E_{1}\left[\xi^{i}\left(t\right)\right]\rangle$, and use this to obtain the first residue, as follows:

(30) $$r_{1}\left(t\right)=\langle M\left(x^{i}\left(t\right)\right)\rangle \;\;\;\;\;i=\left(1,2\ldots S\right)$$

At this point, the first mode at the first stage (i.e., when k = 1) can be obtained as follows:

(31) $$\vec{IMF_{1}}\left(t\right)=x\left(t\right)-r_{1}\left(t\right)$$

Next, to estimate the second residue, white noise is added to the first residue $r_{1}$ as the second local mean $M\langle r_{1}\left(t\right)+\varepsilon_{1}E_{2}\left[\zeta^{i}\left(t\right)\right]\rangle$, and the second mode component is defined as follows:

(32) $$\vec{IMF_{2}}\left(t\right)=r_{1}\left(t\right)-r_{2}\left(t\right)=r_{1}\left(t\right)-\langle M\left(r_{1}\left(t\right)+\varepsilon_{1}E_{2}\left(\xi^{i}\left(t\right)\right)\right)\rangle$$

Furthermore, when stage *k* cannot be decomposed, the kth residual is computed as follows:

(33) $$\vec{IMF_{k}}\left(t\right)=r_{k-1}\left(t\right)-r_{k}\left(t\right)$$

The Hilbert transform is applied to each of the above IMF components separately to obtain the following:

(34) $$c_{i}\left(t\right)=\frac{1}{\pi }\int_{-\infty }^{\infty }\frac{c_{i}\left(\tau \right)}{t-\tau }d\tau$$

Construct a parser function as follows:

(35) $$z_{i}\left(t\right)=c_{i}\left(t\right)+j{\hat{c}}_{i}\left(t\right)=a_{i}\left(t\right)e^{j\varphi_{i}\left(t\right)}$$

Expanding Equation (34) yields the Hilbert spectrum of x(t), denoted as follows:

(36) H(ω,t)=Re∑i=1nai(t)ej∫ωi(t)dt

**Figure 11:**
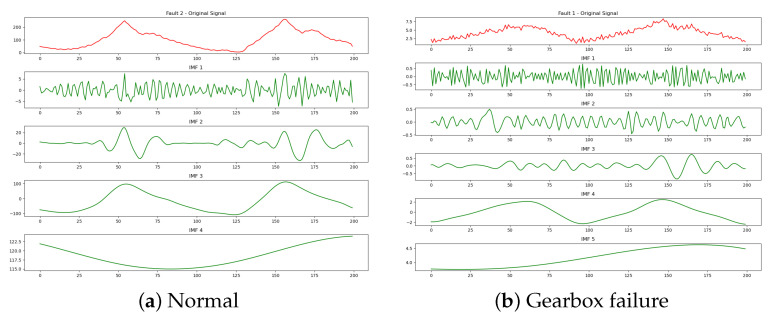
IMF components. (**a**) Normal. (**b**) Gearbox failure.

Then, the Hilbert marginal spectrum is expressed as follows:

(37) $$h\left(\omega \right)=\int_{0}^{T}H\left(\omega ,t\right)dt$$

where *T* is the total signal duration. The Hilbert marginal spectrum h(ω) clearly reveals the distribution of the original signal x(t) based on frequency changes. It reflects how the amplitude of x(t) varies at any given frequency point within the entire frequency distribution. The IMF components and marginal spectra for typical working conditions obtained by the modified Hilbert yellow transform are shown in Figure 11.

**Figure 12:**
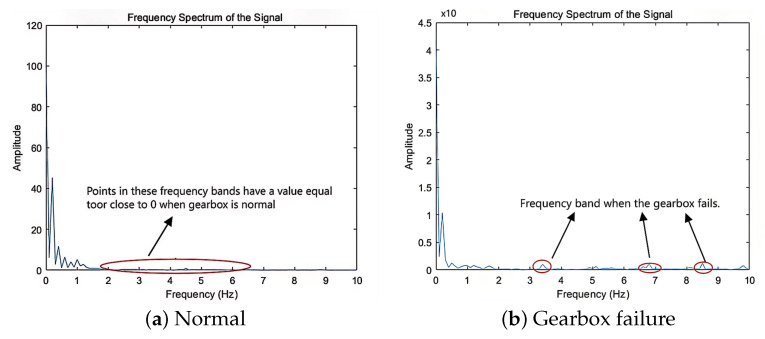
Marginal spectrum. (**a**) Normal. (**b**) Gearbox failure.

Since the sampling period is 50 ms, according to Shannon’s theorem, the marginal spectrum contains only 0–10 Hz signals. This can be seen through the marginal spectrum of Figure 12.

The energy distribution of the electric power diagrams under different working conditions is different, the energy of the low-frequency signals from 0 to 2 Hz mainly originates from the movement of the pumping rods, and the signal components of the higher frequency signals from 2 to 10 Hz are mainly due to the failure of the reduction gearboxes and mechanical vibration caused by the sand production. Although the amplitude of the abnormal frequency is much smaller than the base frequency, long-term wear may waste power. When sand production in the reservoir is severe, it may cause production reduction and shutdown, which may seriously affect the oil well production and cause economic loss. Therefore it is crucial to analyze the electric power signal in time and frequency domain.

The cliff value, which is very sensitive to the vibration signal, is chosen to screen the characteristic signal parameters for determining the vibration, and for a given discrete vibration signal, the cliff coefficient *K* is expressed as follows:

(38) $$K=\frac{1}{N}\sum_{i=1}^{N}{\left(\frac{n_{i}-\bar{n}}{\sigma_{t}}\right)}^{4}$$

where *i* represents the position of the discrete point in the component; $n_{i}$ represents the signal value; $\bar{n}$ represents the signal mean; *N* represents the sampling length; and $\sigma_{t}$ represents the standard deviation.

The crags of all IMF components were found and normalized according to the following quation:

(39) $$\psi_{10}=K_{i}^{\prime }=\frac{K_{i}}{\sum_{i=1}^{m}K_{i}}$$

where *i* represents the IMF component labeling, and *m* represents the number of IMF components.

In addition, the Hilbert marginal spectrum can reflect the cumulative distribution of the energy of the vibration generated at different frequencies during the occurrence of faults. Therefore, frequency features and energy region change features are extracted for the Hilbert marginal spectrum. Three frequency features are defined as follows:

(1) Center of mass frequency (FC):

(40) $$\psi_{11}=FC=\frac{1}{2\pi \Delta f}\frac{\sum_{i}^{N}f_{i}X_{i}}{\sum_{i}^{N}X_{i}}$$

(2) Mean square frequency (MSF):

(41) $$\psi_{12}=MSF=\frac{1}{4\pi^{2}\Delta f^{2}}\frac{\sum_{i}^{N}f_{i}^{2}X_{i}}{\sum_{i}^{N}X_{i}}$$

(3) Frequency variance (VF):

(42) $$\psi_{13}=VF=\frac{1}{4\pi^{2}\Delta f^{2}}\frac{\sum_{i}^{N}{\left(f_{i}-FC\right)}^{2}X_{i}}{\sum_{i}^{N}X_{i}}$$

where *N* is the length of the data, $f_{i}$ is the *i*th frequency value, $X_{i}$ is the amplitude corresponding to $f_{i}$, and Δf is the frequency resolution.

The steps for the energy region change feature extraction are as follows:

Step 1: Observe the results of the change in the energy of the marginal spectral distribution with frequency, and equalize it into five sub-intervals with different energy distributions, according to the range of frequencies.

Step 2: Sum the energies in each sub-interval. Let $E_{i}$ be the sum parameter for the *i*th range interval.

(43) $$E_{i}=\sum_{k=1}^{N}{\left|c_{\mathrm{ik}}\right|}^{2}$$

where $C_{\mathrm{ik}}\left(i=1,2,\cdots n;k=1,2,\cdots N\right)$ represents the amplitude of the *i*th discrete point, and *N* is the number of sampling points.

Step 3: Find the sum parameter of the energy over the entire frequency range interval, given by *E*.

Step 4: Normalize by E normalized to the baseline; the characteristic parameter of the ith sub-interval $X_{i}$ is expressed as follows:

(44) $$X_{i}=E_{i}/E$$

Step 5: The marginal spectrum energy transformation feature vector *X* is given by the following:

(45) $$\psi_{14}=X=\left[X_{1},X_{2},X_{3},X_{4},X_{5}\right]$$

## 4. Fuzzy C-Mean Clustering Effectiveness Evaluation Function

The Fuzzy C-Means (FCM) clustering algorithm is a classical clustering method proposed by [25] and popularized by [26]. The FCM algorithm is widely used in several fields such as highway pavement condition detection [27], image segmentation [28], medical detection [29], and data mining [30]. In this paper, we propose a new FCM clustering effectiveness evaluation function based on multiple clustering performance evaluation modules by combining fuzzy affiliation and datasets characterized by overlapping samples, noisy data, high dimensionality, and a higher number of samples. Firstly, five modules are proposed to define the compactness, overlap, cluster shape, similarity, and separation of the data (as shown in Table 4); then, the clustering validity evaluation function is constructed based on the proposed clustering performance evaluation module, and the minimum value of its function indicates the best clustering result. Finally, the clustering validity function based on the multi-clustering performance evaluation module is used to conduct the simulation experiments on 10 UCI datasets.

Five clustering performance evaluation modules based on tightness, overlap, cluster shape, similarity, and separation definitions constitute a ratio type of clustering validity function defined as follows:

(46) $$V_{\mathrm{LXY}}=\frac{\sum_{i=1}^{c}\sum_{j=1}^{n}{\left(u_{ij}\right)}^{2}{∥x_{j}-v_{i}∥}^{2}+\min_{i\neq j}\left(\frac{1}{n}\sum_{j=1}^{n}\left(1-\left|u_{ik}-u_{jk}\right|\right)\right)+\frac{\lambda_{\max,i}}{\lambda_{\min^{i}}}}{\min_{1\leq i\leq c}\sum_{j=1}^{n}{\left(u_{ij}\right)}^{2}+\min_{i\neq k}{∥v_{i}-v_{k}∥}^{2}+\frac{1}{c}\sum_{i=1}^{c}{∥v_{i}-\bar{v}∥}^{2}}$$

**Figure 13:**
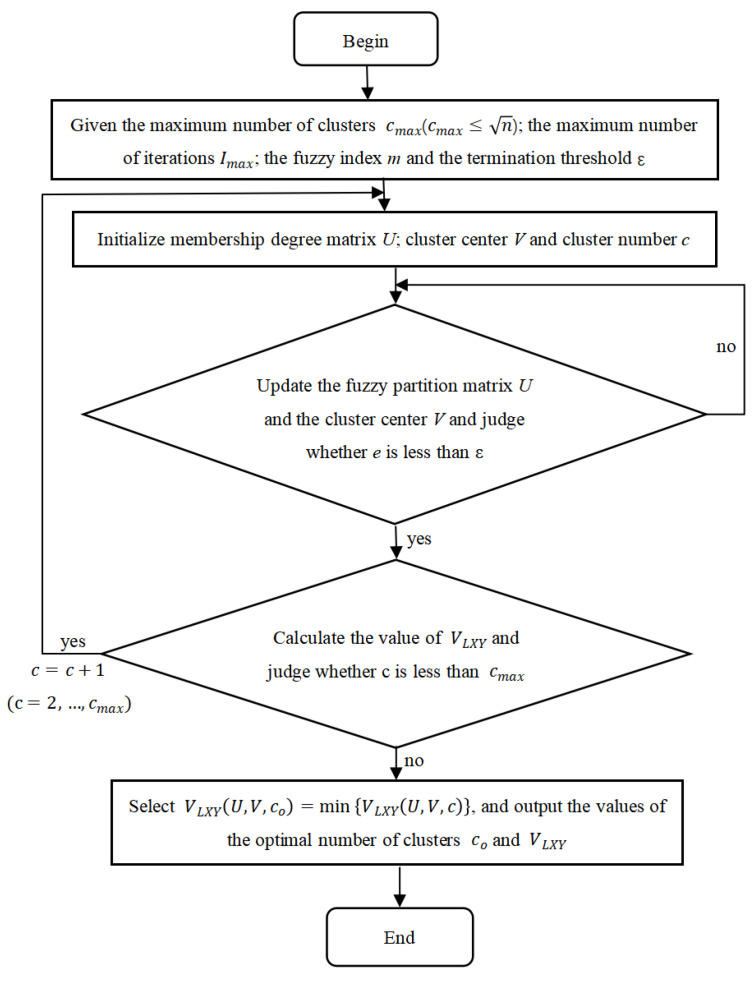
Flowchart of FCM clustering algorithm based on the proposed validity function.

The following section applies $V_{LXY}$ to the FCM clustering algorithm, and the algorithmic flow for obtaining the optimal number of clusters is illustrated in Figure 13.

The process of the FCM clustering algorithm based on $V_{\mathrm{LXY}}$ of FCM clustering algorithm process is as follows:

Step 1: Given the maximum number of clusters $c_{\max}$

$\left(c_{\max}\leq \sqrt{n}\right)$, the maximum number of iterations $I_{\max}$, the fuzzy index m(1.5≤m≤2.5), and the termination threshold ε.

Step 2: Initialize the membership matrix *U*, the cluster center *V* and the number of clusters *c*.

Step 3: Update the fuzzy partition matrix $U^{\left(t+1\right)}$ and cluster center $V^{\left(t+1\right)}$, and judge whether e=vt+1−vt is less than ε. If e<ε, go to Step 4. Otherwise, if e≥ε, then go to Step 2.

Step 4: Let c=c+1, use the FCM clustering algorithm to calculate the minimum value of $V_{LXY}$, and obtain the optimal solution of *c*. If $c<c_{max}$, repeat Step 2. If $c\geq c_{max}$, go to Step 5.

Step 5: Select the number of clusters $min\left\{V_{LXY}\left(U,V,c_{o}\right)\right\}$ corresponding to $c_{o}$ as the optimal number of clusters, and finally, output the value of $V_{LXY}$.

In order to verify the $V_{LXY}$, the effectiveness of clustering on complex datasets, the following simulation experiments are conducted again using the UCI dataset. The real datasets selected for the experiment are the WBC, Ionosphere, Heartstatlog, Haberman, CMC, Jain, Thyroid, Robotnavigation, Seeds, and Diabetes datasets in the UCI database, and the data volume, categories, and attributes of each UCI dataset are shown in Table 5.

The experimental results are analyzed, and when dealing with datasets with overlapping samples, noisy data, high dimensionality, and a higher number of samples, the $V_{LXY}$ ideal number of clusters can be found.

**Figure 14:**
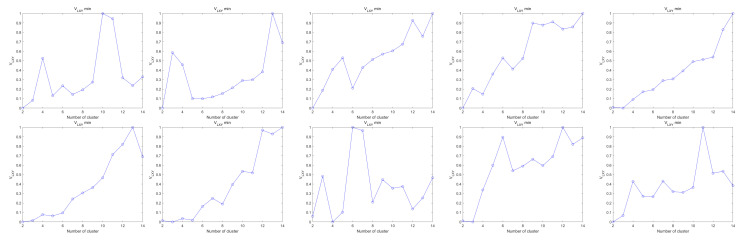
Trends in the clustering validity function.

From the experimental results in Figure 14 and Table 6, it can be seen that $V_{LXY}$, the optimal number of classifications, can be found for the full UCI dataset and that the ideal number of clusters can still be found for datasets with overlapping samples, noisy data, high dimensionality, and a high number of samples.

## 5. Experimental Results

### 5.1. Data Collection

**Figure 15:**
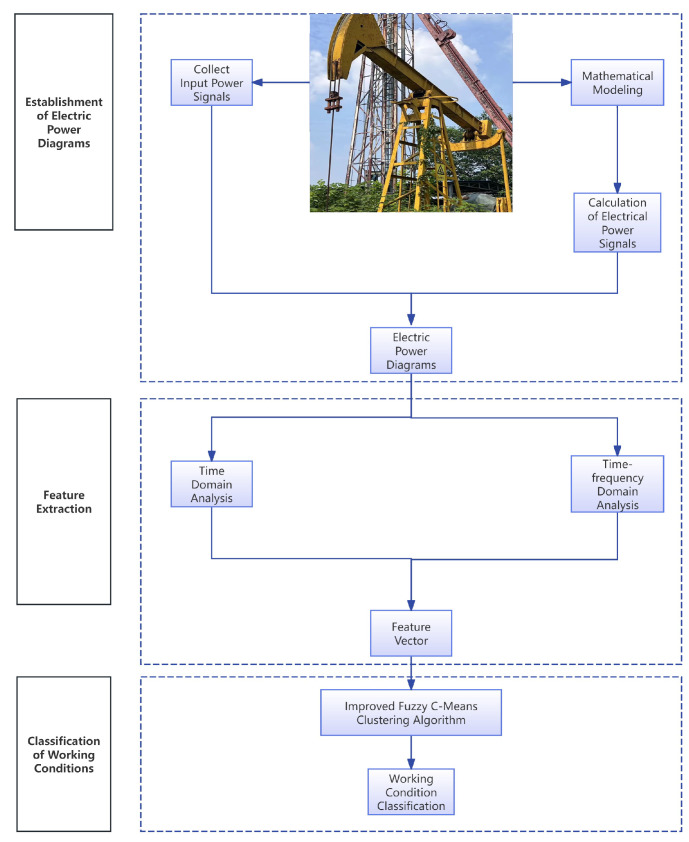
Fault diagnosis flowchart for pumping wells.

**Figure 16:**
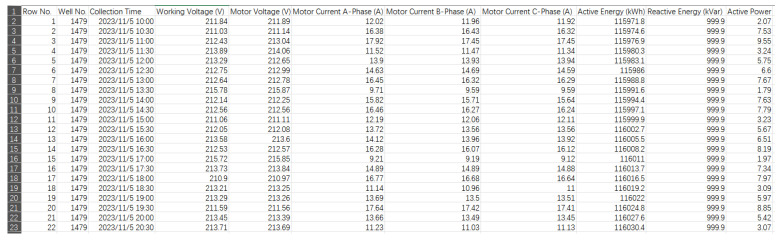
IoT platform for oil and gas production.

In order to prove the effectiveness of the method which is shown in Figure 15, the motor power data and working status information of 128 wells were collected by logging on to the oil and gas production IoT platform of the L oilfield in Xinjiang, as shown in Figure 16.

The on-site electrical measurement system measures and records all electrical parameters, including voltage, current, and phase angle, to analyze both the electrical and mechanical performance of the pumping unit. Its sensors can be permanently installed inside the electrical enclosure and connected via waterproof connectors on the enclosure’s side to a small plug-in radio, enabling wireless communication with the PC station. This setup allows measurements to be taken without opening the electrical enclosure. The TAM software version 2.3 on the PC receives and analyzes the collected data to evaluate key metrics such as power consumption, power generation, pump unit balance, gearbox torque during the upstroke and downstroke, motor load, and power line loss. There are five kinds of motor working statuses as follows: normal, gas influence, insufficient fluid supply, gearbox failure, and sand production. After data cleaning, in order to evaluate the accuracy of the classification results of the unsupervised learning algorithm, the obtained electric power dataset of size 250 is labeled as shown in Table 7.

### 5.2. Feature Extraction

First, the electrical parameter signals are normalized using the maximum–minimum normalization method, which is publicized as follows:

(47) $$p_{k}=\frac{\left(p_{k}-p_{\min}\right)}{\left(p_{\max}-p_{\min}\right)}$$

where $p_{min}$ is the minimum value in the data series; and $p_{max}$ is the maximum value in the data series.

Then, the time domain features are extracted using Equations (20)–(28). Furthermore, the frequency domain and time–frequency domain features are extracted according to Equations (39)–(45). Finally, 14 features are obtained.

### 5.3. Experimental Results

The electric power dataset that has been labeled is clustered using the fuzzy c-clustering validity function algorithm, and in accordance with previous a priori knowledge, it is possible to determine the fuzzy indices 1.5≤m≤2.5 and the number of clusters $2\leq c\leq \sqrt{n}$. In this paper, we choose m=2, 2≤c≤14 to carry out the simulation experiments. Firstly, we judge whether the fuzzy c-clustering validity function can give the best number of clusters on the petroleum dataset, and then, we judge whether its classification is accurate.

**Figure 17:**
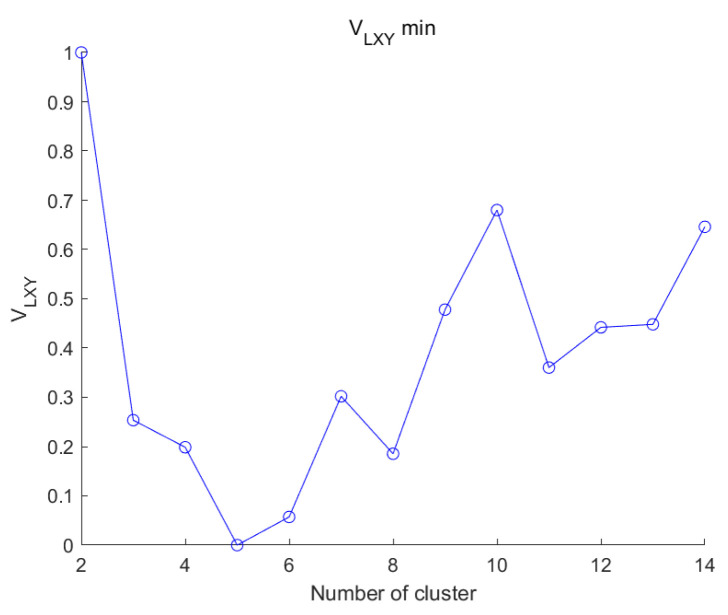
Trends in the clustering validity function.

As shown in Figure 17, $V_{LXY}$ can determine the optimal number of clusters in the dataset.

From Table 8 it can be seen that the classification accuracy of normal and gas influence faults is 100%, the classification accuracy of insufficient fluid supply and sand production is 98%, and the reduction gearbox faults are classified with an accuracy of 96%. Analyzing the classification results, one sample of insufficient fluid supply was wrongly classified as insufficient fluid supply, and three samples were wrongly classified as sand production and gearbox faults. The 98.4% accuracy of the model is accurate and valid because the difference in electric power between insufficient fluid supply and the gas influence in continuous operation of a traveler beam-pumping unit is small, and it is difficult for experienced engineers to give accurate judgments, which is a similar situation with sand production and reduction gearbox failures.

**Figure 18:**
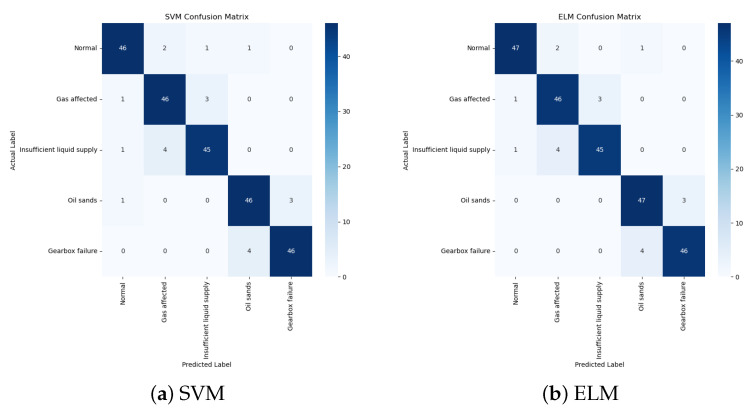
Confusion matrix of SVM and ELM. (**a**) SVM. (**b**) ELM.

SVM and ELM models are selected for comparison due to their strong performance in classification tasks, particularly in the context of fault diagnosis for complex, non-linear systems. SVM can map data to a higher-dimensional space via kernel functions, effectively capturing intricate fault patterns. Meanwhile, ELM uses a single-layer feed-forward network with randomly initialized hidden nodes, offering significantly faster training times while still capturing non-linear relationships. These properties make both models suitable benchmarks for fault diagnosis in this study. To ensure a comprehensive comparison, we rigorously optimized the hyperparameters of the SVM and ELM models. For SVM, a grid search was performed over the regularization parameter C and the kernel coefficient γ, with a quadratic rational kernel selected based on cross-validation performance. For ELM, the number of hidden layer nodes was systematically varied (50–500 nodes), and the sigmoid activation function was adopted to balance nonlinear representation and computational efficiency. The optimal configurations for SVM and ELM were identified through a 10-fold cross-validation on the training dataset. The implementation of SVM relies on the libsvm toolbox. In this study, the SVM model adopts a quadratic rational kernel, a kernel function that can effectively deal with the nonlinear features of the data, and finds the optimal decision boundary by transforming to a higher dimensional space.The ELM model adopts an enhanced stochastic neural network structure, a structure that can quickly deal with the nonlinear features of the data, and effectively improves the training speed and generalization ability of the model by randomly generating hidden layer nodes. The confusion matrices of SVM and ELM are shown in Figure 18. It can be seen that the classification accuracy of SVM in training is 90.8%, and the classification accuracy of ELM is 92%.The test results of the three models are shown in Table 9.

From the analysis of the experimental results, all three methods exhibit high classification accuracy, demonstrating the effectiveness of the multi-feature extraction electrical parameter method proposed in this paper for diagnosing the working conditions of pumping wells. The FCM clustering validity function $V_{LXY}$, based on the multi-cluster performance evaluation module, is capable of identifying the optimal number of clusters when handling UCI datasets and petroleum datasets with overlapping samples, noise, high dimensionality, and a large number of samples. This performance highlights the superiority of the classification model proposed in this paper.

### 5.4. Application in Engineering Practice

The fault diagnosis technology proposed in this paper has high simulation accuracy, but the oilfield production site is complex and has many uncontrollable factors, therefore, in order to verify the effectiveness of this technology in actual production, it is tested in an oil well in Xinjiang.

**Figure 19:**
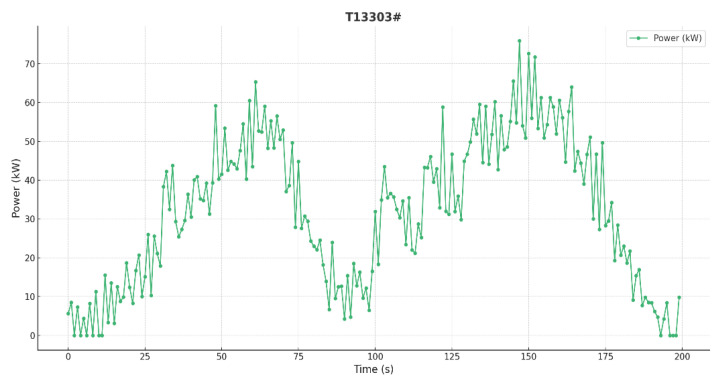
Measured electric work diagram.

The electric power diagram for Well T13303, measured on 3 October 2023, is shown in Figure 19.

The operating condition identification system diagnosed a gearbox failure, prompting maintenance personnel to promptly repair the gearbox. After the equipment was shut down for maintenance, production was resumed. A reduction in power loss was observed, and the post-maintenance electric power diagram indicates normal operation, confirming the effectiveness of this technology in real-world production processes.

## 6. Conclusions

In this paper, a new method for the fault diagnosis of traveling beam-pumping units based on electric power is designed. Due to the small sample of faulty electric power, the dynamic mathematical model of the pumping unit system is first established by considering the geometrical parameters and working parameters of the traveling beam-pumping unit, and the electric power curve is obtained through calculation and compared with the theoretical electric power curve to verify the characteristics of the electric power curve under different working conditions. Then, 14 new features in the time domain, frequency domain, and time–frequency domain are proposed in combination with the working mechanism of the traveling beam-pumping unit, and finally a fuzz c-clustering effectiveness function classification model is established for classification. Subsequently, the proposed method was tested using the electric power dataset from the L oilfield in China. The experimental results show that the new feature extraction method proposed in this paper can accurately characterize the motor power curves under different working conditions, with classification accuracies of over 90% in the FCM, SVM, and ELM models. Additionally, the fuzzy c-means clustering validity function classification model proposed in this paper achieved an accuracy of 98.4%, outperforming the other two methods. Finally, the effectiveness of this method has been demonstrated in engineering practice. The current system exhibits limitations in generalizability across variable real-world operational conditions and scalability to larger datasets or diverse pumping units, with computational efficiency concerns under expanded data volumes. Additionally, its diagnostic accuracy is constrained by the insufficient representation of rare failure modes in training data. Future research should prioritize adaptive modeling techniques for environmental dynamics and fault tolerance, computational optimization for real-time processing at scale, and enhanced failure detection through data augmentation with atypical failure cases or semi-supervised anomaly detection frameworks. Validation should extend to cross-unit applicability and extreme operational scenarios to strengthen robustness.

## Figures and Tables

**Table 1:** Basic parameters of the beam-pumping unit.

Parameter	Symbol	Unit
Crank radius	*R*	m
Connecting rod length	*P*	m
Beam rear arm length	*C*	m
Base rod length	*K*	m
Beam front arm length	*D*	m
Horizontal projection of the base rod	*I*	m

**Table 2:** Basic parameters of the oil well.

Oil-Well Tag	1#	2#	3#	4#	5#
Pumping unit type	CYJ5-2.5-26HB	CYJ5-2.5-26HB	CYJ6-2.5-26HB	CYJ8-3-48HB	CYJ10-3-53HB
Total transmission ratio of the gearbox	34.357	34.357	34.357	30.1875	30.1875
Rated load (KN)	50	50	60	60	100
Rated voltage (V)	380	380	380	380	380
Rated power (KW)	22	22	22	38	37
Beam forearm (mm)	2500	2500	2500	3000	3000
Beam rear arm (mm)	2400	2400	2400	2500	2500
Connecting rod length (mm)	3026	3026	2300	3200	3200
Base pole vertical distance (mm)	2952	2952	3200	3200	3280
Base pole horizontal distance (mm)	2820	2820	2300	2400	2990
Crank radius (mm)	1935	1935	2487	1840	1950
Crank center of gravity radius (mm)	850	850	670	790	625
Single block crank quality (kg)	580	580	750	1772	1740
Single balance weight mass (kg)	700	700	650	990	1075
Structural unbalanced weight (KN)	0.23	0.23	2.85	−0.86	2.25
Motor speed (r/min)	730	730	730	980	980
Oil well conditions	Normal	Gas-affected	Insufficient liquid supply	Oil sands	Gearbox failure

**Table 3:** Evaluation indicators for different oil wells.

Indicator	1#	2#	3#	4#	5#
MSE	0.00605	0.01905	0.01869	0.02905	0.03926
MAE	0.06355	0.07435	0.06974	0.14435	0.17256
RSE	0.18227	0.27938	0.28950	0.43938	0.47262

**Table 4:** Clustering validity evaluation modules.

Modules	Formula	Features of the Module
**Compactness**	$\mathrm{Com}=\sum_{i=1}^{c}\sum_{j=1}^{n}{\left(u_{ij}\right)}^{2}{∥x_{j}-v_{i}∥}^{2}$	Represents the sum of distances between the cluster center $v_{i}$ and the data sample $x_{j}$. A smaller value indicates higher similarity and tighter data within the class.
**Overlap**	$\mathrm{Ove}=\min_{i\neq j}\left(\frac{1}{n}\sum_{j=1}^{n}\left(1-|u_{ik}-u_{jk}|\right)\right)$	Measures the overlap between clusters based on a membership degree. A smaller Ove value indicates better separation of overlapping data.
**Cluster shape**	$\mathrm{Cs}=\frac{\lambda_{\max,i}}{\lambda_{\min,i}}$	The ratio of the maximum eigenvalue ($\lambda_{\max,i}$) to the minimum eigenvalue ($\lambda_{\min,i}$) of the covariance matrix of cluster *i*. Smaller ratios indicate cluster shapes closer to a sphere.
**Similarity**	$\mathrm{Sim}=\min_{1\leq i\leq c}\sum_{j=1}^{n}{\left(u_{ij}\right)}^{2}$	Represents the sum of squares of membership degrees. Larger Sim values indicate higher similarity within the cluster.
**Separation1**	${\mathrm{Sep}}_{1}=\min_{i\neq k}{∥v_{i}-v_{k}∥}^{2}$	Defines the minimum distance between any two cluster centers. Larger Sep_1_ values indicate better separation between clusters.
**Separation2**	${\mathrm{Sep}}_{2}=\frac{1}{c}\sum_{i=1}^{c}{∥v_{i}-\bar{v}∥}^{2}$	Represents the average distance between cluster centers and their equilibrium point. Larger Sep_2_ values indicate better separation between clusters.

**Table 5:** UCI datasets used in component experiments.

Dataset	Data Numbers	Attributes	Classes
WBC	683	9	2
Ionosphere	351	34	2
Heartstatlog	270	13	2
Haberman	306	3	2
CMC	1473	9	3
Jain	373	2	2
Thyroid	215	5	3
Robotnavigation	5456	25	4
Seeds	210	7	3
Diabetes	768	8	2

**Table 6:** Best cluster numbers for different validity functions for the UCI datasets.

Data	WBC	Ionosphere	Heartstatlog	Haberman	CMC	Jain	Thyroid	Robotnavigation	Seeds	Diabetes
Optimal *c*	2	2	2	2	3	2	3	4	3	2
$V_{LXY}$	2	2	2	2	3	2	3	4	3	2

**Table 7:** Oil dataset details.

Data	Number of Samples	Serial Number
Normal	50	0–49
Gas affected	50	50–99
Insufficient liquid supply	50	100–149
Oil sands	50	150–199
Gearbox failure	50	200–249

**Table 8:** Test results summary.

Data	Accuracy	Number of Samples	Serial Number
Normal	100%	50	0–49
Gas affected	100%	50	50–99
Insufficient liquid supply	98%	50	100–149
Oil sands	98%	50	150–199
Gearbox failure	96%	50	200–249

**Table 9:** Comparison of results for different methods.

Model	FCM	SVM	ELM
Accuracy	0.9840	0.9080	0.9200

## Data Availability

Data are contained within the article.
